# To Be or Not to Be Expressed: The First Evidence of a Nucleolar Dominance Tissue-Specificity in *Brachypodium hybridum*

**DOI:** 10.3389/fpls.2021.768347

**Published:** 2021-12-06

**Authors:** Natalia Borowska-Zuchowska, Ewa Robaszkiewicz, Serhii Mykhailyk, Joanna Wartini, Artur Pinski, Ales Kovarik, Robert Hasterok

**Affiliations:** ^1^Plant Cytogenetics and Molecular Biology Group, Institute of Biology, Biotechnology and Environmental Protection, Faculty of Natural Sciences, University of Silesia in Katowice, Katowice, Poland; ^2^Department of Molecular Epigenetics, Institute of Biophysics, Academy of Sciences of the Czech Republic, Brno, Czechia

**Keywords:** nucleolar dominance, 35S rDNA, secondary constriction, *Brachypodium*, allopolyploidy, 3D-FISH, rRNA gene expression

## Abstract

Nucleolar dominance (ND) is an epigenetic, developmentally regulated phenomenon that describes the selective inactivation of 35S rDNA loci derived from one progenitor of a hybrid or allopolyploid. The presence of ND was documented in an allotetraploid grass, *Brachypodium hybridum* (genome composition DDSS), which is a polyphyletic species that arose from crosses between two putative ancestors that resembled the modern *B. distachyon* (DD) and *B. stacei* (SS). In this work, we investigated the developmental stability of ND in *B. hybridum* genotype 3-7-2 and compared it with the reference genotype ABR113. We addressed the question of whether the ND is established in generative tissues such as pollen mother cells (PMC). We examined condensation of rDNA chromatin by fluorescence *in situ* hybridization employing state-of-art confocal microscopy. The transcription of rDNA homeologs was determined by reverse-transcription cleaved amplified polymorphic sequence analysis. In ABR113, the ND was stable in all tissues analyzed (primary and adventitious root, leaf, and spikes). In contrast, the 3-7-2 individuals showed a strong upregulation of the S-genome units in adventitious roots but not in other tissues. Microscopic analysis of the 3-7-2 PMCs revealed extensive decondensation of the D-genome loci and their association with the nucleolus in meiosis. As opposed, the S-genome loci were always highly condensed and localized outside the nucleolus. These results indicate that genotype-specific loss of ND in *B. hybridum* occurs probably after fertilization during developmental processes. This finding supports our view that *B. hybridum* is an attractive model to study ND in grasses.

## Introduction

Allopolyploidy is an interspecific hybridization followed by chromosome doubling and it is believed to play an essential role in angiosperm evolution (Mason and Wendel, 2020). The genomic investigations of many plant allopolyploids of different evolutionary ages have revealed that the newly formed allopolyploid is subjected to immediate (short-term) and long-term changes that operate at both genomic and transcriptomic levels (Wendel et al., 2018). The short-term consequences of allopolyploidy, including meiotic irregularities, chromosomal aberrations, transposon proliferation, widespread loss of non-coding sequences, and gene expression alterations, may constitute a considerable challenge for most newly formed allopolyploids (Ozkan et al., 2001; Shaked et al., 2001; Nicolas et al., 2009; Chester et al., 2012; Yoo et al., 2014). However, once they stabilize over a longer evolutionary timeframe, allopolyploids may be characterized by an increased phenotypic variability compared to their diploid progenitors and, therefore, may have a higher capacity to reach new environmental niches (Soltis et al., 2015).

The tandemly repeated 35S rRNA genes have been the subject of many studies on plant hybrids and allopolyploids, particularly for inferring the molecular background of nucleolar dominance (ND; previously known as “differential amphiplasty”), a widespread phenomenon that was initially documented by Navashin (1934) in *Crepis* hybrids. In the allopolyploids and hybrids that exhibit ND, the 35S rDNA loci that are inherited from one progenitor are dominant over the others (Pikaard, 2000; Volkov et al., 2004; Ge et al., 2013; Symonova, 2019). It is well known that ND is maintained in an epigenetic manner and that the under-dominant rDNA loci are hypermethylated, especially within the rRNA gene promoters (Matyasek et al., 2016) and are characterized by repressive epigenetic marks, e.g., the dimethylated lysine 9 of histone H3 (H3K9me2) (Neves et al., 1995; Chen and Pikaard, 1997a; Houchins et al., 1997; Lawrence et al., 2004; Borowska-Zuchowska and Hasterok, 2017). Moreover, it was shown that the inactivation of the rRNA gene loci is accompanied by an RNA-dependent DNA methylation pathway (RdDM) (Preuss et al., 2008; Costa-Nunes et al., 2010) and the deacetylation of the histones (Earley et al., 2006). However, the question of how specific rDNA loci are selected for inactivation still remains unanswered.

Nucleolar dominance is a common phenomenon among grass allopolyploids, including the economically important cereals, e.g., bread wheat (*Triticum aestivum* L.) (Cermeno et al., 1984; Guo and Han, 2014) and triticale (Lacadena et al., 1984; Vieira et al., 1990; Neves et al., 1995). Therefore, most molecular and cytogenetic research concerning the molecular basis of ND among monocots has been limited to wheat and its derivatives. Considering the massive genome size of wheat of ∼16.6 Gbp/1C (Dolezel et al., 2018), which is composed of three subgenomes (2*n* = 42; genome composition AABBDD) in which many 35S rDNA loci are present, studies of ND in this species are tremendously complicated. Thus, it is necessary to find a monocot model system that will be amenable in ND studies.

In 2008, the presence of ND was observed in the root-tip cells of a small- and simple-genome allotetraploid grass, *Brachypodium hybridum* (2*n* = 30; DDSS). Its putative ancestral species are two diploids that resembled the modern *B. distachyon* (2*n* = 10; DD) and *B. stacei* (2*n* = 20; SS). It was documented using silver staining followed by fluorescence *in situ* hybridization (FISH) with 25S rDNA as the probe that the S-genome-derived 35S rDNA loci are not transcribed in the root-tip meristematic cells (Idziak and Hasterok, 2008). The further analyses of the ND in *B. hybridum* were mainly focused on the reference genotype ABR113. Cytomolecular studies that employed immunostaining approaches revealed that the D- and S-genome-inherited 35S rDNA loci were differentiated by distinct epigenetic patterns. The D-genome-originated loci were characterized by significantly lower DNA methylation levels compared to the S-genome ones and were enriched in the acetylated isoforms of histones, e.g., H4K5ac, H4K16ac, and H3K9ac. By contrast, the S-genome loci were usually located at the nuclear periphery and were enriched in the heterochromatic H3K9me2 mark (Borowska-Zuchowska and Hasterok, 2017). Studies on the developmental regulation in ABR113 did not reveal any stage in *B. hybridum* ontogenesis in which the ND is abolished (Borowska-Zuchowska et al., 2016, 2019). We recently observed a reduction in the S-genome-like 35S rDNA copy number in 16 *B. hybridum* genotypes. *B. stacei-*derived rDNA contributions to total rDNA varied from 7 to 39%, which suggests that the inactive loci may have gradually been eliminated during evolution (Borowska-Zuchowska et al., 2020). Such a variation in ancestral rDNA ratios may be related to the polyphyletic origin of *B. hybridum*. Recent studies of plastomes, nuclear single nucleotide variants, and k-mers associated with retrotransposons showed two independent origins for *B. hybridum* (∼1.4 and ∼0.14 million years ago; Gordon et al., 2020). Considering that *B. hybridum* has only one 35S rDNA locus per ancestral genome and that the whole genomic sequence of both putative ancestors and several *B. hybridum* genotypes have been published (IBI, 2010; Gordon et al., 2020), this allotetraploid grass may become a suitable model in the ND studies in monocots. However, such a model should also provide a wide range of genotypes in which there is a diversity in the rDNA structure and expression. In the current study, we applied a combination of cytogenetic and molecular methods in order to verify whether the expression status of the 35S rDNA homeologs is additive or biased at the various developmental stages in *B. hybridum* and revealed a genotype in which there is a differential expression of S-genome 35S rRNA genes in some organs.

## Materials and Methods

### Plant Material

Two genotypes of *Brachypodium hybridum* were used in this study. The reference genotype ABR113 (Portugal) was obtained from the Institute of Biological, Environmental and Rural Sciences (Aberystwyth University, Aberystwyth, United Kingdom). The 3-7-2 genotype was derived from the T_1_ generation of the germplasm that had been collected in Turkey (geographical location: N38°17′40.2″ E27°24′13.9″; altitude: 173 m). All of the plants were grown in pots with soil mixed with vermiculite (3:1, w/w) at 20–22°C and a 16 h photoperiod in a greenhouse.

To analyze mitotic metaphase chromosomes from the primary roots, dehusked seeds were grown on a filter paper that had been moistened with tap water for 3 days at room temperature in the dark. Whole seedlings with 1.5-3-cm-long roots were immersed in ice-cold water for 24 h and then fixed in methanol:glacial acetic acid (3:1, v/v) at 4°C overnight and stored at −20°C. Immature spikes for the analysis of the meiocytes were collected from 2 to 2.5-month-old plants, fixed in fresh ethanol:glacial acetic acid (3:1, v/v) at room temperature. The fixative was replaced by a fresh one after 24 h. The material was stored at −20°C until use.

### DNA Isolation and Southern Blot Hybridization

Total genomic DNAs (gDNAs) from the leaves from 1.5-month-old plants of *B. hybridum* were isolated using a CTAB buffer as described previously (Borowska-Zuchowska et al., 2020). The purified gDNAs (1 μg/sample) were digested with the methylation insensitive *Bgl*II restriction enzyme cutting the AGATCT motif. The digested gDNAs were separated by gel electrophoresis on 1% (w/v) agarose gel and alkali-blotted onto nylon membranes (Hybond XL, GE Healthcare Life Sciences). The membranes were hybridized with ^32^P-labeled DNA probes (DekaLabel kit, ThermoFisher Scientific) using the ∼220 bp-long PCR product derived from the 3′ end of the 25S rDNA of tobacco as previously described (Kovarik et al., 2005). The hybridization bands were visualized with a phosphorimager (Typhoon 9410, GE Healthcare), and the radioactivity of the bands was quantified with ImageQuant software (GE Healthcare). The ancestral rDNA proportions were expressed as a percentage of signal in 6.7 kb (*B. distachyon*-specific band) or 7.8 kb + 9.2 kb (*B. stacei*-specific bands) fractions out of the total (6.7 kb + 7.8 kb + 9.2 kb) rDNA signal. At least two individuals (two biological replicates) from each *B. hybridum* genotype were analyzed.

### Fluorescence *in situ* Hybridization on the Squashed Mitotic Preparations

The root meristem cytogenetic preparations, including the multi-substrate preparations, were made as described (Hasterok et al., 2006; Jenkins and Hasterok, 2007). A 2.3-kb *Cla*I subclone of the 25S rDNA of *A. thaliana* (Unfried and Gruendler, 1990) was used as the probe to detect the 35S rDNA loci. The clone was labeled with tetramethylrhodamine-5-dUTP (Roche) *via* nick translation (Hasterok et al., 2002). Fluorescence *in situ* hybridization (FISH) was performed as previously described (Idziak et al., 2011). After precipitation, the 25S rDNA probe was dissolved in a hybridization mixture containing 50% deionized formamide and 20% dextran sulfate in 2 × saline sodium citrate buffer (SSC). The hybridization mixture was pre-denatured at 75°C for 10 min and applied to the chromosome preparations. After denaturation at 75°C for 4.5 min, the preparations were allowed to hybridize in a humid chamber at 37°C for 16–20 h. Post-hybridization washes were performed in 10% formamide in 0.1 × SSC at 42°C (79% stringency). The chromosomes were mounted and counterstained in Vectashield (Vector Laboratories) containing 2.5 μg ml^–1^ of 4′,6-diamidino-2-phenylindole (DAPI; Serva). Three different individuals (three biological replicates) have been analyzed for both primary and adventitious roots. Only the metaphases with visible secondary constrictions on the D-genome chromosomes have been considered (15–26 metaphases/meristem). In the case of interphase nuclei, approx. 100 nuclei have been analyzed for each individual.

### Fluorescence *in situ* Hybridization on the Polyacrylamide Pads

The meiocytes of *B. hybridum* were embedded in acrylamide gel to preserve their three-dimensional architecture. The procedure of embedding was adopted from Bass et al. (1997). Briefly, fixed anthers were collected into a 1 × Buffer A (2 × buffer A salts, 1 mM DTT, 0.2 mM spermine, 0.5 mM spermidine, 0.35 M sorbitol) and macerated using a brass tapper. Next, 10 μl of the meiocyte suspension was transferred onto a 24 × 24 mm coverslip, mixed with 5 μl of activated acrylamide stock, and immediately covered with another coverslip. After 1 h of polymerization, the coverslips were separated using a razor blade. The one containing the polyacrylamide pad was transferred to a Petri dish and the FISH reaction was performed.

FISH on the polyacrylamide pads was performed as was described in Borowska-Zuchowska et al. (2019). A pre-denatured hybridization mixture with 25S rDNA as the probe was applied to the pads, and they were denatured together at 75°C for 8 min. After renaturation (∼20 h), post-hybridization washes were performed in 20% formamide in 0.1 × SSC at 37°C. The pads were mounted in a mounting medium [1 μg ml^–1^ DAPI, 200 mM Tris-HCl pH 8, 2.3% DABCO (1,4-diazobicyclo(2,2,2)octane) and 78% glycerol] and stored at 4°C until the images were acquired.

### Image Acquisition and Analysis

Images of the mitotic metaphase chromosomes after FISH were acquired using a Zeiss Axio Imager.Z.2 wide-field fluorescence microscope equipped with an AxioCam HRm monochromatic camera. The meiocytes that had been embedded in the polyacrylamide gel were optically sectioned using an Olympus FV1000 confocal microscope system equipped with a 60×/1.35 PlanApo objective. All of the image stacks were acquired by scanning from the top to the bottom of a meiocyte in 0.25 μm steps and then processed using MBF ImageJ (Wayne Rasband, National Institutes of Health, Bethesda, MD, United States).

### 35S rDNA Expression Analysis

The procedures followed those described by Borowska-Zuchowska et al. (2020). Briefly, the total RNA was isolated from (i) 1.5-3-cm-long primary roots that had been collected from 3-day-old seedlings (three biological replicates); (ii) fresh, greenish leaves and adventitious roots that had been collected from 1.5-month-old plants (three biological replicates); and (iii) immature spikes that had been collected from 2 to 2.5-month-old plants (two biological replicates). The RNA was isolated using a NucleoSpin^®^ RNA Plant and Fungi kit (Macherey-Nagel). Any contaminating DNA was removed using an RNase-Free DNase set (Qiagen). Each reverse transcription reaction contained 1 μg of total RNA and 1 μl of Maxima H Minus Enzyme Mix (Thermo Fisher Scientific) and was performed according to the manufacturer’s instructions. The obtained cDNAs were used as templates in the ITS1 amplification using PCR with the primers 18S and 5.8S rev (Kovarik et al., 2005). The ITS1 PCR products were subjected for restriction with *Mlu*I enzyme for 2 h at 37°C and separated using gel electrophoresis.

## Results and Discussion

### The Structure of 35S rDNA Loci in *Brachypodium hybridum* 3-7-2

**FIGURE 1:**
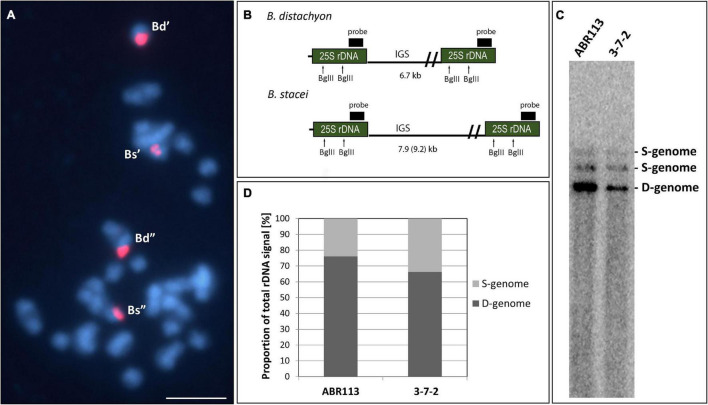
The structure of the 35S rRNA gene loci in *B. hybridum* genotype 3-7-2. **(A)** FISH mapping of 25S rDNA (red fluorescence) in the highly condensed metaphase chromosomes of the genotype 3-7-2. Chromatin was stained with DAPI (blue fluorescence). Scale bar = 5 μm. **(B)** The *Bgl*II restriction maps in progenitor *B. distachyon* and *B. stacei* rDNA units. For simplicity, only the 25S rRNA genes are shown. **(C)** Southern blot hybridization of the genomic DNAs from the genotypes 3-7-2 and ABR113 (control) subjected to restriction with *Bgl*II. The blot was hybridized with a 220-bp-long fragment of 25S rDNA. **(D)** The quantification of 35S rDNA homeologs. The ancestral rDNA contributions are denoted as the proportion of the D- or S-genome-specific signals to the total rDNA signal.

The FISH method with 25S rDNA as the probe was used to analyze the 35S rRNA gene loci number and chromosomal localization in the 3-7-2 genotype of *B. hybridum*. The analysis involved the meristematic cells from both, the primary and adventitious roots. Because the S- and D-genome-derived 35S rDNA loci occupy distinct and different positions on the chromosomes, no additional chromosome markers are required to differentiate the loci from the two ancestors. In our previous works, we showed that the *B. distachyon*-originated 35S rDNA locus is located on the terminal part of the short arm of chromosome Bd5, while the *B. stacei*-inherited locus occupies the proximal region of the significantly smaller Bs10 chromosome (Hasterok et al., 2004; Idziak and Hasterok, 2008; Borowska-Zuchowska et al., 2016; Lusinska et al., 2018). FISH revealed that the 3-7-2 genotype has two chromosomal pairs that bear 35S rDNA loci, one from each progenitor (Figure 1A). Their chromosomal positions were the same as in the reference genotype ABR113.

Our previous study showed that the intensity of the relative *B. stacei*-like 35S rDNA FISH signal corresponded to the S-genome rDNA contribution to the total rDNA, which was quantified based on the Southern blot hybridization results (Borowska-Zuchowska et al., 2020). To verify ancestral contributions of 35S rDNA, Southern blot hybridization with 25S rDNA as a probe was performed. The gDNAs from 3-7-2 and ABR113 (as a control) were digested with the *Bgl*II restriction enzyme. As was shown previously, there are two recognition sites for *Bgl*II in the 25S rDNA of both D- and S-genome rDNA units (Figure 1B; Borowska-Zuchowska et al., 2020). In Figure 1C, the 25S rDNA probe hybridized to the three *Bgl*II fragments in DNAs from both ABR113 and 3-7-2: (i) a fast-migrating, 6.7-kb-long fragment representing the D-genome rDNA units; (ii) 7.9-kb-long fragment representing the S-genome rDNA units in which both *Bgl*II sites were cut by the enzyme; and (iii) a slow-migrating fragment representing the full-size S-genome rDNA unit. The radioactivity of the hybridization bands was estimated by a phosphorimager revealing the quantitative relationships between the ancestral rDNAs (Figure 1D). The contributions of the *B. stacei*-derived rDNA units to the total rDNA were 33.8% and 23.9% in 3-7-2 and ABR113, respectively. In line with this observation, the FISH hybridization signals corresponding to the 35S rDNA loci from the S-genome were more prominent in 3-7-2 than their counterparts in ABR113 (Figure 1A; data for ABR113 have already been published: Borowska-Zuchowska et al., 2016). Thus, the 3-7-2 genotype possesses a higher number of S-genome rDNA units than ABR113.

So far, the *B. stacei*-derived rDNA contributions to total rDNA vary from 7% to 39% among different *B. hybridum* genotypes (Borowska-Zuchowska et al., 2020). Since *B. hybridum* is a polyphyletic species that arose from multiple crosses between the two ancestors during Quaternary (Catalan et al., 2012; Diaz-Perez et al., 2018; Gordon et al., 2020), the fate of the rDNA homeologs in the different genotypes of this allotetraploid may be accompanied by various evolutionary scenarios. Therefore, the gradual elimination of the *B. stacei-*inherited rDNA units may be still in progress in some genotypes.

### Nucleolar Dominance Is Stable in Leaves but Not in Roots of *Brachypodium hybridum*

**FIGURE 2:**
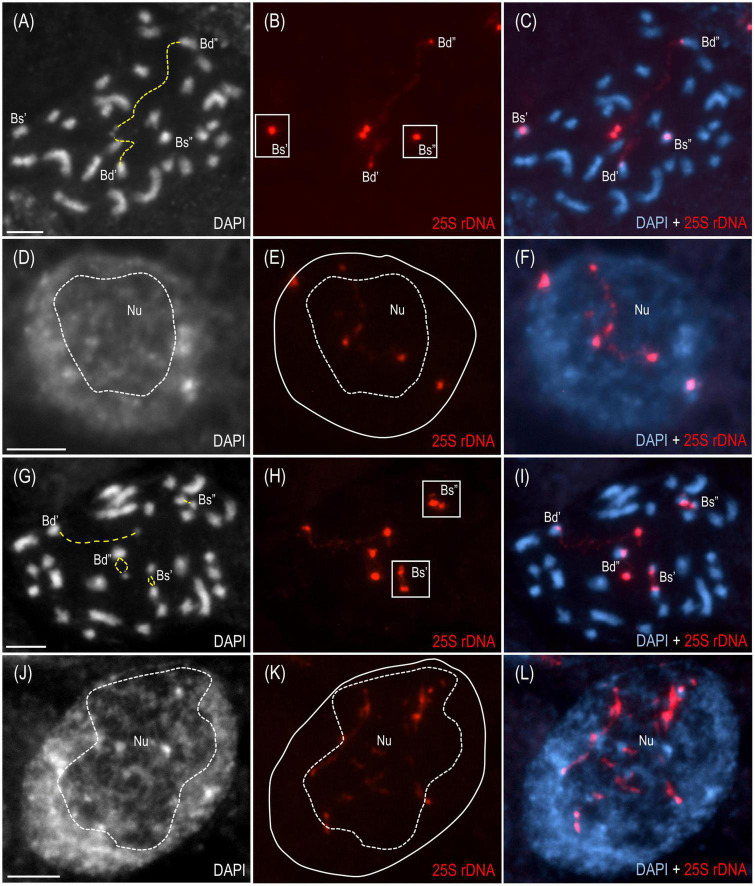
The distribution of the 35S rDNA loci in the mitotic metaphase chromosomes and interphase nuclei of *B. hybridum* genotype 3-7-2. The cells originating from the primary **(A–F)** and adventitious **(G–L)** root apical meristems are presented.. FISH mapping of 25S rDNA (red fluorescence) in the metaphase chromosome complements **(A–C,G–I)** and interphase nuclei **(D–F,J–L)**. Bd, *B. distachyon*-inherited 35S rDNA loci; Bs, *B. stacei*-inherited 35S rDNA loci; Nu, nucleolus. The secondary constrictions on **(A,G)** are indicated by the yellow dashed lines. The position of the nucleolus on **(E,K)** is denoted by a white dashed line. Chromatin was stained with DAPI. Scale bar = 5 μm.

The cytogenetic and molecular approaches were applied across four different tissues of *B. hybridum* 3-7-2 and, to the best of our knowledge, revealed that the ND in this species may be developmentally regulated for the first time. Because only the transcriptionally active 35S rDNA loci can form secondary constrictions on the metaphase chromosomes (Shaw, 2013), the presence of these structures constitutes indirect proof of the 35S rDNA activity. Thus, in the current studies, we verified whether the D- and S-genome 35S rDNA loci colocalized with the secondary constrictions on the chromosomes from the primary (Figures 2A–C and Supplementary Figures 1A–C) and adventitious roots of *B. hybridum* 3-7-2 (Figures 2G–I and Supplementary Figure 2) using FISH with 25S rDNA as a probe. Only the D-genome 35S rDNA loci formed secondary constrictions in the primary roots, while the *B. stacei*-inherited ones remained highly condensed (Figures 2A–C and Supplementary Figures 1A–C). Interestingly, all of the ancestral 35S rDNA loci formed secondary constrictions in the adventitious root-tip cells (Figures 2G–I and Supplementary Figure 2). Thus, ND was not stable in this type of roots. These observations were further corroborated at the level of the interphase nuclei from the root-tip cells. As was shown by FISH, only one pair of 35S rDNA loci corresponding to the D-genome was located adjacent to the nucleolus in the primary roots, whereas the *B. stacei*-like loci occupied the nuclear periphery and were located in the DAPI-positive, heterochromatic domains (Figures 2D–F and Supplementary Figures 1D–F). By contrast, all of the 25S rDNA FISH signals were located adjacent to or within the nucleolus in the interphase nuclei from the adventitious roots (Figures 2J–L and Supplementary Figures 2A–C), which strongly suggests that all of the 35S rDNA loci contributed to the formation of the nucleolus.

**FIGURE 3:**
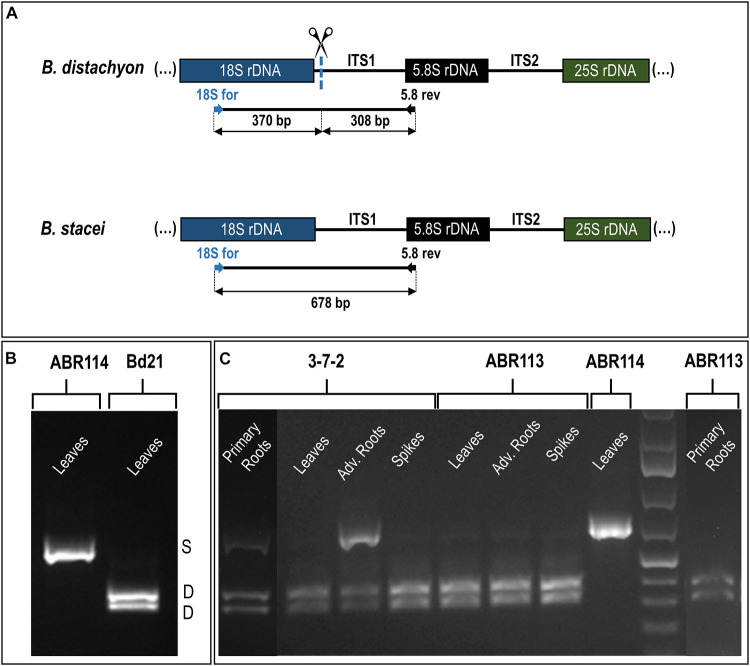
The 35S rDNA expression analysis in the different tissues of *B. hybridum* (genotypes 3-7-2 and ABR113) and *B. stacei* (genotype ABR114; control) and *B. distachyon* (genotype Bd21; control) using the RT-PCR CAPS method. **(A)** The *Mlu*I restriction profile of the *B. distachyon-*like and *B. stacei-*like ITS1 PCR products. The expected sizes of the bands after *Mlu*I digestion are presented. **(B)** The *Mlu*I restriction profiles of ITS1 amplification products that were obtained from the leaves cDNAs of *B. distachyon* and *B. stacei*. **(C)** The *Mlu*I restriction profiles of ITS1 amplification products that were obtained from the primary roots, leaves, adventitious roots, and immature spikes cDNAs. Adv. Roots, adventitious roots.

The reverse-transcription cleaved amplified polymorphic sequence (RT-CAPS) approach was used to examine the expression of the 35S rDNA homeologs at the different developmental stages of *B. hybridum*. The analysis involved RNA from the vegetative organs (primary and adventitious roots and leaves) and the generative organs (immature spikes; discussed further in the next paragraph). Because of the ITS1 sequence divergence between the two ancestral species, the rRNA precursors (pre-rRNA) that originated from the D- and S-genome 35S rDNA loci could be identified. The *Mlu*I enzyme only cuts the *B. distachyon*-like units producing two fragments (Figures 3A,B). Because there was no *Mlu*I restriction site in the *B. stacei*-like ITS1, its RT-PCR products were uncut (Figures 3A,B). In ABR113, only the D-genome *Mlu*I fragments were observed (the presence of two bands per each gel lane; Figure 3C), which indicated a strong uniparental silencing of the S-genome rRNA genes in all of the studied organs. However, in *B. hybridum* 3-7-2, both the D- and S-genome bands were present in the primary and adventitious roots, which implies a co-dominance of the ancestral rDNAs (Figure 3C). The lower intensity of the S-genome band in the sample from the primary root compared with that of the adventitious root was most probably correlated with the lower expression level of the *B. stacei*-inherited rDNA. The banding pattern in leaves and immature spikes of 3-7-2 indicated the expression dominance of the D-genome 35S rDNA (Figure 3C).

A tissue-specific expression pattern of the rDNA homeologs has been observed in many plant hybrids and allopolyploids (Volkov et al., 2007). For instance, in *Arabidopsis suecica*, the progressive silencing of the *A. thaliana*-originated rRNA genes occurred during the early postembryonic development in the tissues that had been derived from both the shoot and root apical meristems (Pontes et al., 2007). Although a fully established ND was observed in leaves, a trace expression of the *A. thaliana*-derived rRNA genes was detected in the root-tip cells of the mature *A. suecica* plants. Similarly, in several cultivars of the allotetraploid *Brassica napus*, the *B. oleracea-*inherited rDNA was stably repressed in the leaves (except for “Norin 9” cultivar, in which a co-dominance of ancestral rDNA was revealed in leaves and roots; Chen and Pikaard, 1997b; Sochorova et al., 2017). In the 2–3-day-old seedlings of *B. napus*, however, there was an expression of both ancestral rDNAs in the root-tip cells (Hasterok and Maluszynska, 2000b). Considering these observations and the current study, it can be concluded that the ND in natural allopolyploids is much more stable in the leaf tissue than in the roots. However, the establishment of ND may occur as early as 4–5 days after fertilization as was shown by silver staining in wheat-rye hybrids (Castilho et al., 1995). Thus, the mechanisms that determine the ancestral rDNA expression status at different ontogenetic stages may vary significantly between species.

To date, all of the studied *B. hybridum* genotypes have shown a strong, uniparental dominance toward the D-genome 35S rDNA in the roots and leaves as was revealed using the cytogenetic (Idziak and Hasterok, 2008; Borowska-Zuchowska et al., 2016) and molecular approaches (Borowska-Zuchowska et al., 2020). It is well known from many plant systems, however, that epigenetic mechanisms are behind the ND maintenance, making this phenomenon a reversible process (Vieira et al., 1990; Chen and Pikaard, 1997a; Lawrence et al., 2004; Earley et al., 2006; Preuss et al., 2008). Early studies using chemical agents that cause the hypomethylation of the genome (e.g., 5-azacytidine; 5-aza-2’-deoxycytidine) and/or histone deacetylation (trichostatin A) resulted in the transcriptional reactivation of the under-dominant rDNA loci (Vieira et al., 1990; Amado et al., 1997; Chen and Pikaard, 1997a; Lawrence et al., 2004). By contrast, the global hypomethylation of the *B. hybridum* ABR113 genome induced by 5-azacytidine was insufficient for the transcriptional reactivation of the S-genome loci (Borowska-Zuchowska and Hasterok, 2017). This observation suggested that the *B. stacei*-inherited rRNA genes in ABR113 may be irreversibly repressed, e.g., due to the accumulation of mutations. Thus, further investigations on the diverse *B. hybridum* genotypes are needed to find those that are characterized by a differential expression of the ancestral rDNA homeologs. In this study, a co-dominant expression of both ancestral rDNA loci in *B. hybridum* was revealed in the root tissue of the 3-7-2 genotype for the first time. Interestingly, such a pattern was not uniform across the whole root of this genotype. The cytological observation of highly condensed S-genome loci (Figures 2A–C and Supplementary Figure 1) and the absence of significant S-genome transcripts (Figure 3) indicate that the apical meristem of the primary root may not represent a tissue with impaired ND and that considerable rRNA silencing may occur in these cells. In contrast, loss of ND (codominant expression phenotype) seems to be highly pronounced in adventitious roots, which show secondary constrictions on both D- and S-type chromosomes (Figures 2G–I and Supplementary Figure 2) and strong expression of both homeologs (Figure 3). The differential expression of rDNA could be related to the different origins of primary and adventitious roots. In monocots, the primary root derives from an embryonic radicle and is often short-lived and replaced by adventitious roots, which are formed from any non-root tissue, usually stem, and are produced during normal development (Bellini et al., 2014). Previous analyses of Hasterok and Maluszynska (2000a) on *Allium cepa* showed that the 35S rRNA gene expression pattern can differ between the primary and adventitious roots. In their study, ND was manifested in adventitious and not primary roots. In contrast to the present study, in *A. cepa* more rDNA loci were transcriptionally active in the root-tip cells of the primary roots than in the adventitious ones, suggesting that the rRNA gene expression patterns might be species-specific.

### Nucleolar Dominance Is Maintained in the Generative Organs of *Brachypodium hybridum*

**FIGURE 4:**
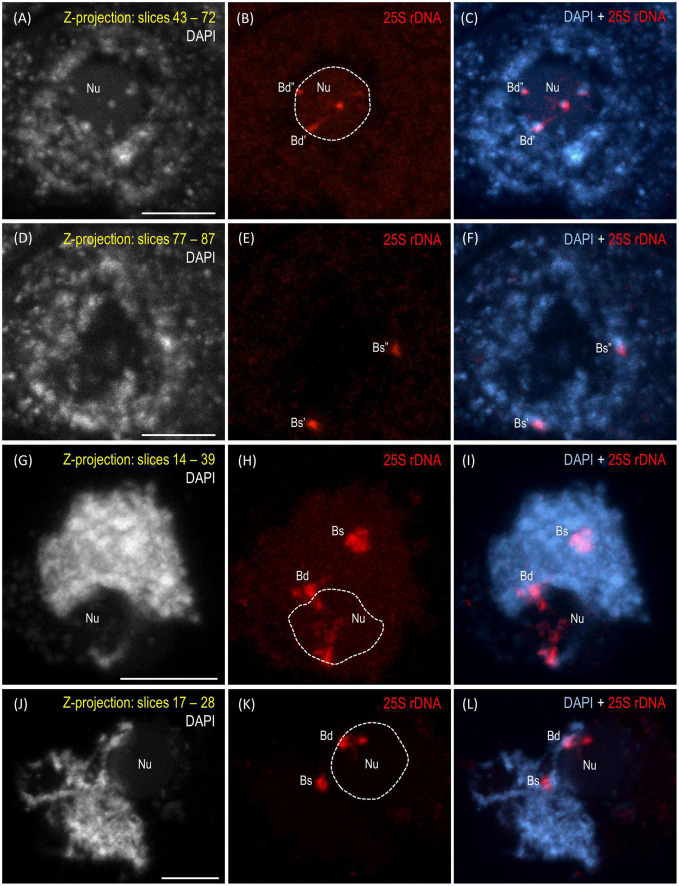
Localization of the *B. distachyon*- and *B. stacei*-originated 35S rDNA loci in 3-D cytogenetic preparations of *B. hybridum* meiocytes (genotype 3-7-2) at leptotene, zygotene, and pachytene. Selected stacks that contain the 25S rDNA hybridization signals (red fluorescence) are presented. **(A–F)** Two different sections of one nucleus at leptotene. **(G–I)** Zygotene. **(J–L)** Zygotene/Pachytene. Bd, *B. distachyon*-inherited 35S rDNA loci; Bs, *B. stacei*-inherited 35S rDNA loci; Nu, nucleolus. The position of the nucleolus in the 25S rDNA channel **(B,E,H,K)** is denoted by a dashed line. Chromatin was counterstained with DAPI. Scale bar = 5 μm.

**FIGURE 5:**
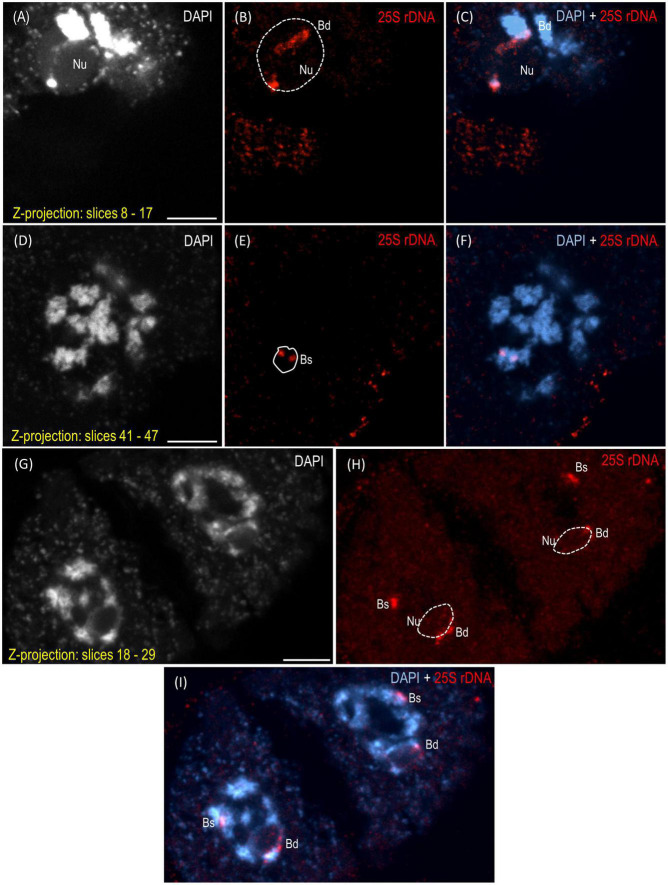
Localization of the *B. distachyon*- and *B. stacei*-originated 35S rDNA loci in the 3-D cytogenetic preparations of *B. hybridum* meiocytes (genotype 3-7-2) at diakinesis and in dyads. Selected stacks that contain the 25S rDNA hybridization signals (red fluorescence) are presented. **(A–F)** Two different sections of one nucleus at diakinesis. **(G–I)** Dyad. Bd, *B. distachyon*-inherited 35S rDNA loci; Bs, *B. stacei*-inherited 35S rDNA loci; Nu, nucleolus. The position of the nucleolus in the 25S rDNA channel **(B,E,H)** is denoted by a dashed line. Chromatin was counterstained with DAPI. Scale bar = 5 μm.

The reprogramming of the rRNA gene expression may accompany the transition from the vegetative to the generative phase as was shown in *Brassica* (Chen and Pikaard, 1997b) and *Solanum* allopolyploids (Komarova et al., 2004). To verify whether such a transcriptional activation of the S-genome 35S rDNA loci occurs in the genotype 3-7-2 of *B. hybridum*, we determined (i) the localization of both ancestral homeologs in the meiocytes that had been isolated from the anthers and (ii) the origin of the pre-rRNA in the immature spikes. The FISH with 25S rDNA as the probe was performed on the *B. hybridum* 3-7-2 meiocytes at different substages of prophase I and in the dyads. Only one nucleolus per cell was observed in all of the studied stages of meiosis (Figures 4, 5 and Supplementary Videos 1–5). At the leptotene, two 35S rDNA loci of a D-genome origin were located within the nucleolus (Figures 4A–C and Supplementary Video 1), while the *B. stacei-*inherited loci were located at the nuclear periphery in the DAPI-positive domains (Figures 4D–F and Supplementary Video 1). Beginning with the zygotene to the latter substages of prophase I, only one 25S rDNA FISH signal per ancestral genome was observed after the formation of the bivalents. One bivalent with decondensed 35S rDNA loci that had been derived from the D-genome was associated with the nucleolus, while an S-genome bivalent with proximally located 35S rDNA loci was not attached to the nucleolus in the meiocytes at the stages from the zygotene to the diakinesis (Figures 4G–L, Figures 5A–F and Supplementary Videos 2–4). Moreover, only one 25S rDNA FISH signal was seen adjacent to/within the nucleolus in the dyads, which indicates that ND was maintained in this phase (Figures 5G–I and Supplementary Video 5). RT-CAPS analysis showed only the D-genome pre-rRNA precursors in the immature spikes of *B. hybridum* 3-7-2 (Figure 3C). Thus, ND was maintained not only in the meiocytes but in all of the floral organs. The exclusion of under-dominant S-genome 35S rDNA loci from the nucleolus contrasts with studies in *Arabidopsis thaliana*, whose all loci, irrespective of activity, seem to associate with the nucleolus in meiosis (Sims et al., 2021). Thus, it cannot be ruled out that the position of NORs is influenced by the nuclear topology, which may differ between diploid and allopolyploid species.

The studies of Silva et al. (1995) in the hexaploid triticale showed that the rRNA genes of a rye origin that were silent during the first meiotic division were transcriptionally activated in the microspores (Silva et al., 1995), thereby indicating that meiotic reprogramming may erase the preferential inactivation of rDNA via ND. Chen and Pikaard (1997b) used an S1 nuclease protection assay to determine the rRNA gene expression patterns in the different tissues of the allotetraploid *B. napus*. They observed the reactivation of the *B. oleracea*-inherited rDNA loci in all of the floral organs, including the sepals, petals, anthers, and siliques. Thus, the hypothesis that a derepression of the under-dominant rRNA genes occurs when both ancestral rDNA homeologs are segregated by meiosis as was shown previously in triticale (Silva et al., 1995) may not be universal for all plant allopolyploids (Reeder, 1985). The further studies of Sochorova et al. (2017) in *B. napus* showed only a trace expression of the *B. oleracea*-derived rDNA in the flower buds of two of the seven studied cultivars. These observations indicated that the ND regulation might even be genotype-specific. Such a specificity of the ND regulation among the floral organs in *B. hybridum* has not been observed yet. In addition to the current study on genotype 3-7-2, the ND establishment in the meiocytes was only analyzed in the reference genotype ABR113 (Borowska-Zuchowska et al., 2019). The S-genome rDNA homeologs could not form the nucleolus in either of the studied genotypes as they were transcriptionally silenced. The inactive state of the aforementioned loci in ABR113 was further confirmed using the silver staining method (Borowska-Zuchowska et al., 2019).

## Conclusion and Perspectives

**FIGURE 6:**
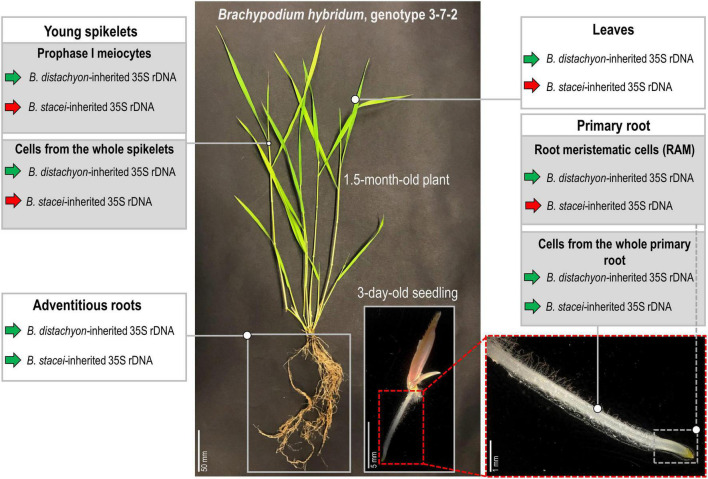
Stability of the nucleolar dominance in different tissues of *B. hybridum* genotype 3-7-2. Green arrows denote the transcriptionally active 35S rDNA, while the red arrows indicate the silenced ones.

To the best of our knowledge, this study revealed the first *B. hybridum* genotype with a co-dominance of the D- and S-genome rDNA homeologs in the primary and adventitious roots. Figure 6 summarizes the rDNA expression patterns in the different tissues of *B. hybridum* 3-7-2 and shows the developmental regulation of ND in this species. Further comparative studies of the rDNA molecular structure in *B. hybridum* may shed more light on the specific mechanisms that shape ND in grasses. There is some evidence that both the chromosomal position and/or the presence of the control elements that are located within rDNA units may be responsible for the preferential expression of rDNA *via* ND (Chandrasekhara et al., 2016; Mohannath et al., 2016). Thus, using the new whole-genome sequencing strategies that permit a closer look at the complete rDNA units (McKinlay et al., 2021) may significantly improve our understanding of the rDNA evolution and behavior in allopolyploids.

## Data Availability Statement

The original contributions presented in the study are included in the article/Supplementary Material; further inquiries can be directed to the corresponding author.

## Author Contributions

NB-Z conceived and designed the study. NB-Z, ER, SM, JW, AP, and AK performed the experiments. NB-Z, ER, AK, and RH analyzed the data. NB-Z and RH wrote the manuscript. All authors contributed to the article and approved the submitted version.

## Conflict of Interest

The authors declare that the research was conducted in the absence of any commercial or financial relationships that could be construed as a potential conflict of interest.

## Publisher’s Note

All claims expressed in this article are solely those of the authors and do not necessarily represent those of their affiliated organizations, or those of the publisher, the editors and the reviewers. Any product that may be evaluated in this article, or claim that may be made by its manufacturer, is not guaranteed or endorsed by the publisher.

## Supplementary Material

Supplementary Figure 1 The rDNA-FISH analysis of primary root apical meristems of *B. hybridum* genotype 3-7-2. The distribution of the 35S rDNA loci in the mitotic metaphase chromosomes and interphase nucleus is shown in **A–F**, respectively. FISH mapping of 25S rDNA (red fluorescence) in the metaphase chromosome complement **(A–C)** and interphase nucleus **(D–F)**. Bd, *B. distachyon*-inherited 35S rDNA loci; Bs, *B. stacei*-inherited 35S rDNA loci. The secondary constrictions on **(A)** are indicated by the dashed lines. Chromatin was stained with DAPI. Scale bar = 5 μm. Note the absence of secondary constrictions on the Bs chromosomes.

Supplementary Figure 2 The rDNA-FISH analysis of adventitious root apical meristems of *B. hybridum* genotype 3-7-2. The distribution of the 35S rDNA loci in the primary mitotic metaphase chromosomes and interphase nuclei is shown in **(A–F)**. FISH mapping of 25S rDNA (red fluorescence) in the metaphase chromosome complement **(A–F)** and interphase nucleus (**A–C**, left side of the photomicrograph). Bd, *B. distachyon*-inherited 35S rDNA loci; Bs, *B. stacei*-inherited 35S rDNA loci. The secondary constrictions on **(A,D)** are indicated by the dashed lines. Chromatin was stained with DAPI. Scale bar = 5 μm. Note the presence of secondary constrictions on both Bs and Bd loci.

Supplementary Video 1 Three-dimensional distribution of the 25S rDNA hybridization signals (green) in *B. hybridum* 3-7-2 meiocyte at leptotene that is presented in Figures 4A–F. The cell shown in the figure is identified by a white rectangle. Chromatin is pseudocolored in red.

Supplementary Video 2 Three-dimensional distribution of 25S rDNA hybridization signals (green) in *B. hybridum* 3-7-2 meiocyte at zygotene that is presented in Figures 4G–I. Chromatin is pseudocolored in red.

Supplementary Video 3 Three-dimensional distribution of 25S rDNA hybridization signals (green) in *B. hybridum* 3-7-2 meiocyte at zygotene/pachytene that is presented in Figures 4J–L. Chromatin is pseudocolored in red.

Supplementary Video 4 Three-dimensional distribution of 25S rDNA hybridization signals (green) in *B. hybridum* 3-7-2 meiocyte at diakinesis that is presented in Figures 5A–F. Chromatin is pseudocolored in red.

Supplementary Video 5 Three-dimensional distribution of 25S rDNA hybridization signals (green) in *B. hybridum* 3-7-2 dyad that is presented in Figures 5G–I. Chromatin is pseudocolored in red.
