# Corrigendum: To Be or Not to Be Expressed: The First Evidence of a Nucleolar Dominance Tissue-Specificity in *Brachypodium hybridum*

**DOI:** 10.3389/fpls.2022.917940

**Published:** 2022-04-28

**Authors:** Natalia Borowska-Zuchowska, Ewa Robaszkiewicz, Serhii Mykhailyk, Joanna Wartini, Artur Pinski, Ales Kovarik, Robert Hasterok

**Affiliations:** ^1^Plant Cytogenetics and Molecular Biology Group, Institute of Biology, Biotechnology and Environmental Protection, Faculty of Natural Sciences, University of Silesia in Katowice, Katowice, Poland; ^2^Department of Molecular Epigenetics, Institute of Biophysics, Academy of Sciences of the Czech Republic, Brno, Czechia

**Keywords:** nucleolar dominance, 35S rDNA, secondary constriction, *Brachypodium*, allopolyploidy, 3D-FISH, rRNA gene expression

In the original article, there was a mistake in Figure 3B as published. The gel lines were marked in an incorrect order. The corrected Figure 3B appears below.

**Figure 3:**
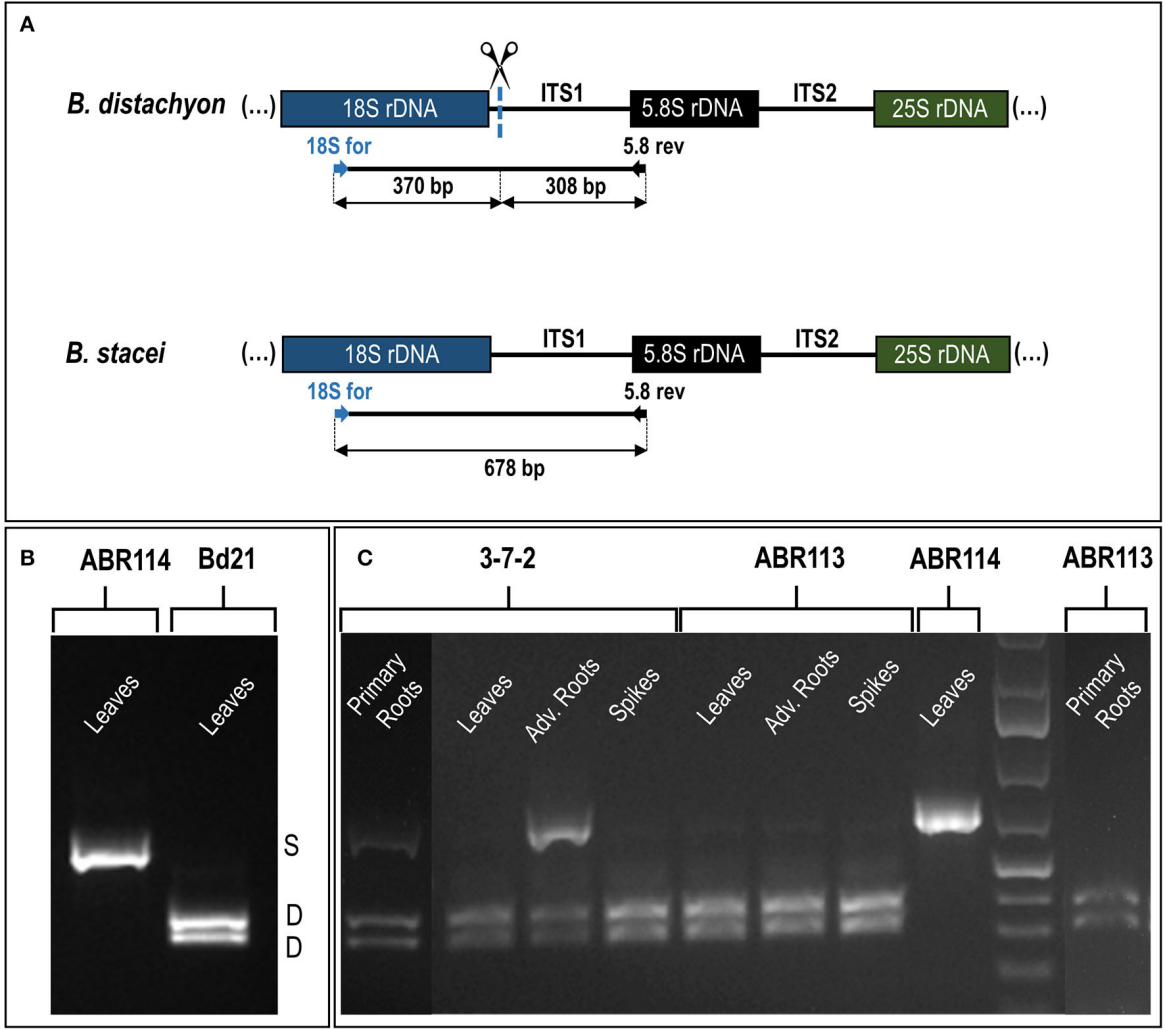
The 35S rDNA expression analysis in the different tissues of *B. hybridum* (genotypes 3-7-2 and ABR113) and *B. stacei* (genotype ABR114; control) and *B. distachyon* (genotype Bd21; control) using the RT-PCR CAPS method. **(A)** The *Mlu*I restriction profile of the *B. distachyon*-like and *B. stacei*-like ITS1 PCR products. The expected sizes of the bands after *Mlu*I digestion are presented. **(B)** The *Mlu*I restriction profiles of ITS1 amplification products that were obtained from the leaves cDNAs of *B. distachyon* and *B. stacei*. **(C)** The *Mlu*I restriction profiles of ITS1 amplification products that were obtained from the primary roots, leaves, adventitious roots, and immature spikes cDNAs. Adv. Roots, adventitious roots.

The authors apologize for this error and state that this does not change the scientific conclusions of the article in any way. The original article has been updated.

## Publisher's Note

All claims expressed in this article are solely those of the authors and do not necessarily represent those of their affiliated organizations, or those of the publisher, the editors and the reviewers. Any product that may be evaluated in this article, or claim that may be made by its manufacturer, is not guaranteed or endorsed by the publisher.

