# Supplementary figures and images for: To Be or Not to Be Expressed: The First Evidence of a Nucleolar Dominance Tissue-Specificity in Brachypodium hybridum
Source: Front Plant Sci. 2021 Dec 6;12:768347. doi: 10.3389/fpls.2021.768347 (PMC8685274; doi:10.3389/fpls.2021.768347)

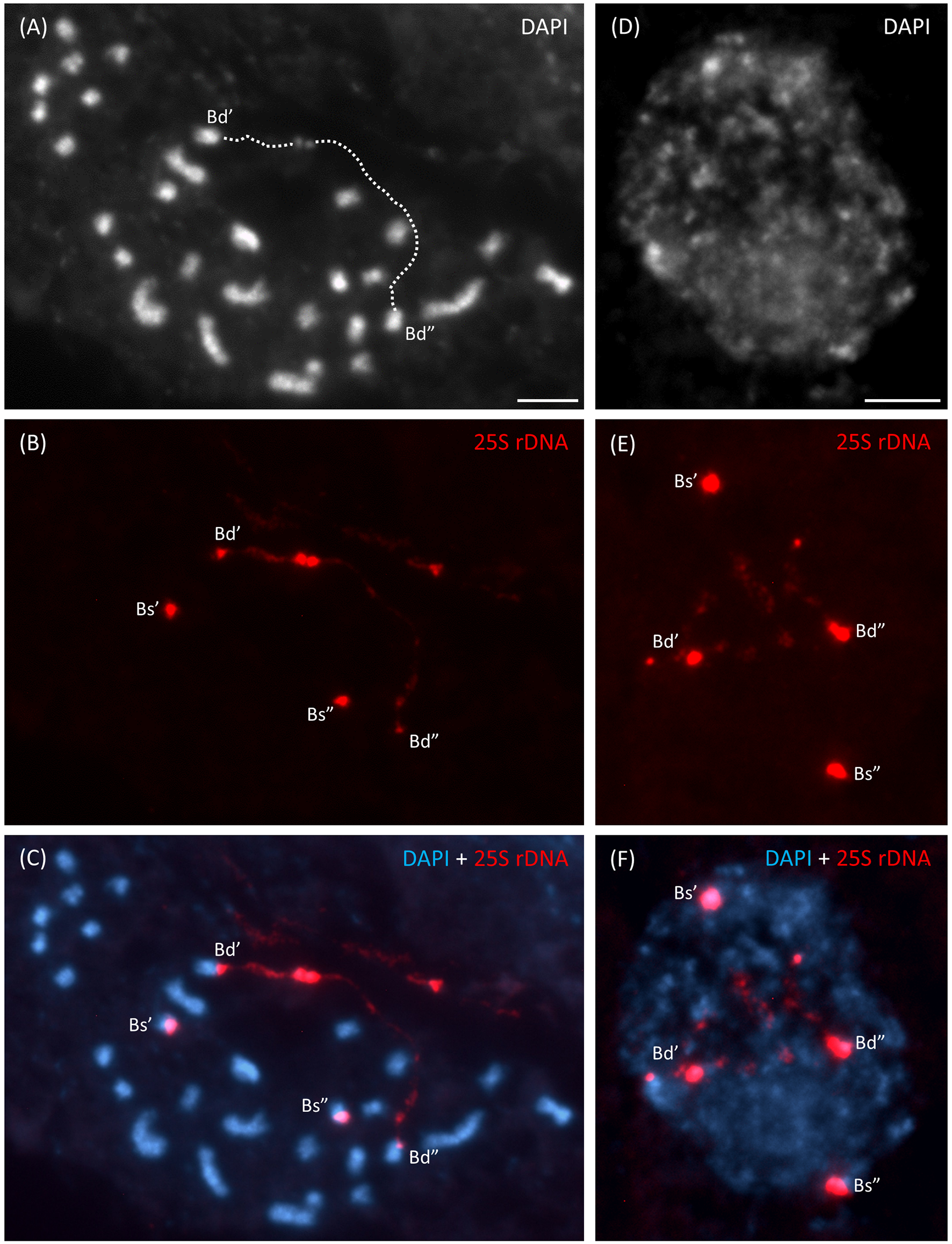

Supplement: Supplementary Figure 1 — The rDNA-FISH analysis of primary root apical meristems of B. hybridum genotype 3-7-2. The distribution of the 35S rDNA loci in the mitotic metaphase chromosomes and interphase nucleus is shown in A–F, respectively. FISH mapping of 25S rDNA (red fluorescence) in the metaphase chromosome complement (A–C) and interphase nucleus (D–F). Bd, B. distachyon-inherited 35S rDNA loci; Bs, B. stacei-inherited 35S rDNA loci. The secondary constrictions on (A) are indicated by the dashed lines. Chromatin was stained with DAPI. Scale bar = 5 μm. Note the absence of secondary constrictions on the Bs chromosomes. [file Image_1.TIF]

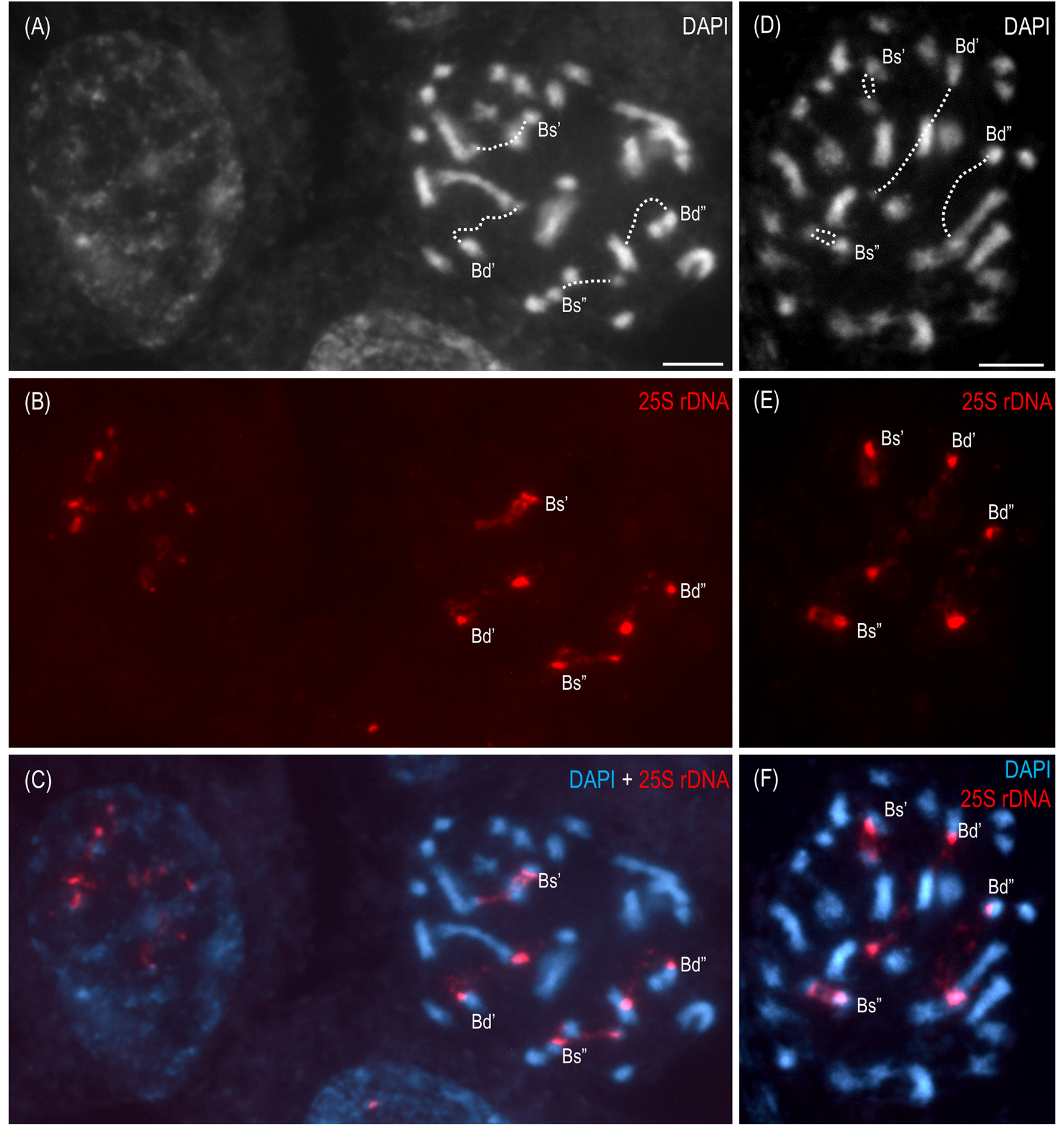

Supplement: Supplementary Figure 2 — The rDNA-FISH analysis of adventitious root apical meristems of B. hybridum genotype 3-7-2. The distribution of the 35S rDNA loci in the primary mitotic metaphase chromosomes and interphase nuclei is shown in (A–F). FISH mapping of 25S rDNA (red fluorescence) in the metaphase chromosome complement (A–F) and interphase nucleus (A–C, left side of the photomicrograph). Bd, B. distachyon-inherited 35S rDNA loci; Bs, B. stacei-inherited 35S rDNA loci. The secondary constrictions on (A,D) are indicated by the dashed lines. Chromatin was stained with DAPI. Scale bar = 5 μm. Note the presence of secondary constrictions on both Bs and Bd loci. [file Image_2.TIF]
